# Impact of Design on Medical Device Safety

**DOI:** 10.1007/s43441-019-00022-4

**Published:** 2019-12-09

**Authors:** Teodora Miclăuş, Vasiliki Valla, Angeliki Koukoura, Anne Ahlmann Nielsen, Benedicte Dahlerup, Georgios-Ioannis Tsianos, Efstathios Vassiliadis

**Affiliations:** Evnia Group, Copenhagen Business Center, Hellerup Strandvejen 60, 2900 Copenhagen, Denmark

**Keywords:** Medical device, Human factors, Design control, Risk management, Ergonomics, Usability engineering

## Abstract

The growing number of emerging medical technologies and sophistication of modern medical devices (MDs) that improve both survival and quality of life indexes are often challenged by alarming cases of vigilance data cover-up and lack of sufficient pre- and post-authorization controls. Combining Quality with Risk Management processes and implementing them as early as possible in the design of MDs has proven to be an effective strategy to minimize residual risk. This article aims to discuss how the design of MDs interacts with their safety profile and how this dipole of intended performance and safety may be supported by Human Factors Engineering (HFE) throughout the Total Product Life-Cycle (TPLC) of an MD in order to capitalize on medical technologies without exposing users and patients to unnecessary risks.

## Introduction

Medical devices (MDs) constitute an inextricable element of the modern healthcare edifice [1–8]. Yet, in this ever-growing and ever-evolving universe of MD industry, performance and expedited approval processes often appear to be enjoying more attention to the detriment of safety concerns and proper reporting of vigilance data [9–16]. Nevertheless, the introduction of medical technology innovation should not forfeit or jeopardize the safety of users and patients. An MD Manufacturer is expected to provide a product that performs as intended throughout its total product life-cycle (TPLC), therefore the need to scrutinize multiple aspects of a device’s design is continuous. At the core of this process lie paramount questions about the user of the MD, the context of use, as well as the risk–benefit profile of the device that should always remain in favor of the user and—first and foremost—of the intended patient [17–20].

The complexity of the laborious process that will take an MD from ‘sketch’ level to its use in a real-life healthcare system is eloquently described by Hollnagel [21], who likens modern healthcare settings to cognitive systems that will not survive unless a functional interaction of humans and technology is achieved. In other words, we are currently in a position where we have to smoothly amalgamate two counterparts that were not necessarily made to co-exist. On the one hand, the MD industry serves its pivotal role by introducing sophisticated new technologies while trying to maintain its revenues amid alarming cases of vigilance data cover-up and lack of sufficient pre- and post-authorization controls [9–11, 22–24]. On the other hand, the physician grapples with the expanding technical demands of medical practice, thus complicating his everyday tasks and increasing medical errors [25–27]. Incorporation of minimally invasive & robotic devices, combinatorial products, use of software and telecommunications in medical practice is technically challenging and requires a very different skillset than that expected from a medical doctor 50 years ago. The modern physician must have a proficient technical dexterity, spatial awareness, and the ability to rapidly integrate information deriving from multiple user-interfaces into his decisions [28, 29]. To cut this Gordian knot, Authorities are struggling to set up a harmonized regulatory framework that will, as much as possible, allow a timely, integrated identification and communication of potential hazards, risks and adverse events, eventually resulting in MD-related risk mitigation [30–41].

Within this context, studying MD usability and spotlighting the essential contribution of MD design has become crucial for patient safety. The increasing need to ensure safety of both patients and healthcare professionals, as well as the effective and efficient use of every MD, has led to the introduction of Human Factors Engineering (HFE) principles and methods into the process of MD design and development. As with other aspects, such as the use of checklists in operating rooms (ORs) [42] healthcare has taken a page from aviation in implementing HFE to MDs; initially following the release of the International Electrotechnical Commission (IEC) standard 62366 in 2007, followed by its recognition by the FDA [43] and introduction of HFE into the European MD Directive requirements [44–46] and, more recently, into the European MD Regulations [47].

This article aims to discuss how the design of MDs impacts their safety profile and how Authorities regulate this dipole of intended performance and safety throughout an MD’s TPLC. Furthermore, we aim to explain why HFE has penetrated the MD field and how it can be implemented to capitalize on innovative, emerging medical technologies without exposing users and patients to unnecessary risks.

## Importance of Medical Device Design

Design and development of an MD are the two most crucial phases of its TPLC because a poorly designed device will not make its way through regulatory compliance into the market; in the unlikely event that it does, failure to safely perform as intended will undermine conformity with Essential Requirements (ERs).

Some degree of risk is obviously inherent to the use of any MD and this risk’s acceptability level is often conditioned by the stakeholders’ own perception of risk, cultural diversity, educational proficiency, and patients’ profile [48–52]. Therefore, understanding how users will interact with the MD within their environment is vital for good design. As such, during the design stage, the first thorough control of an MD is implemented as part of the Quality Management System (QMS) requirements [37, 53–58].

**Figure 1.**
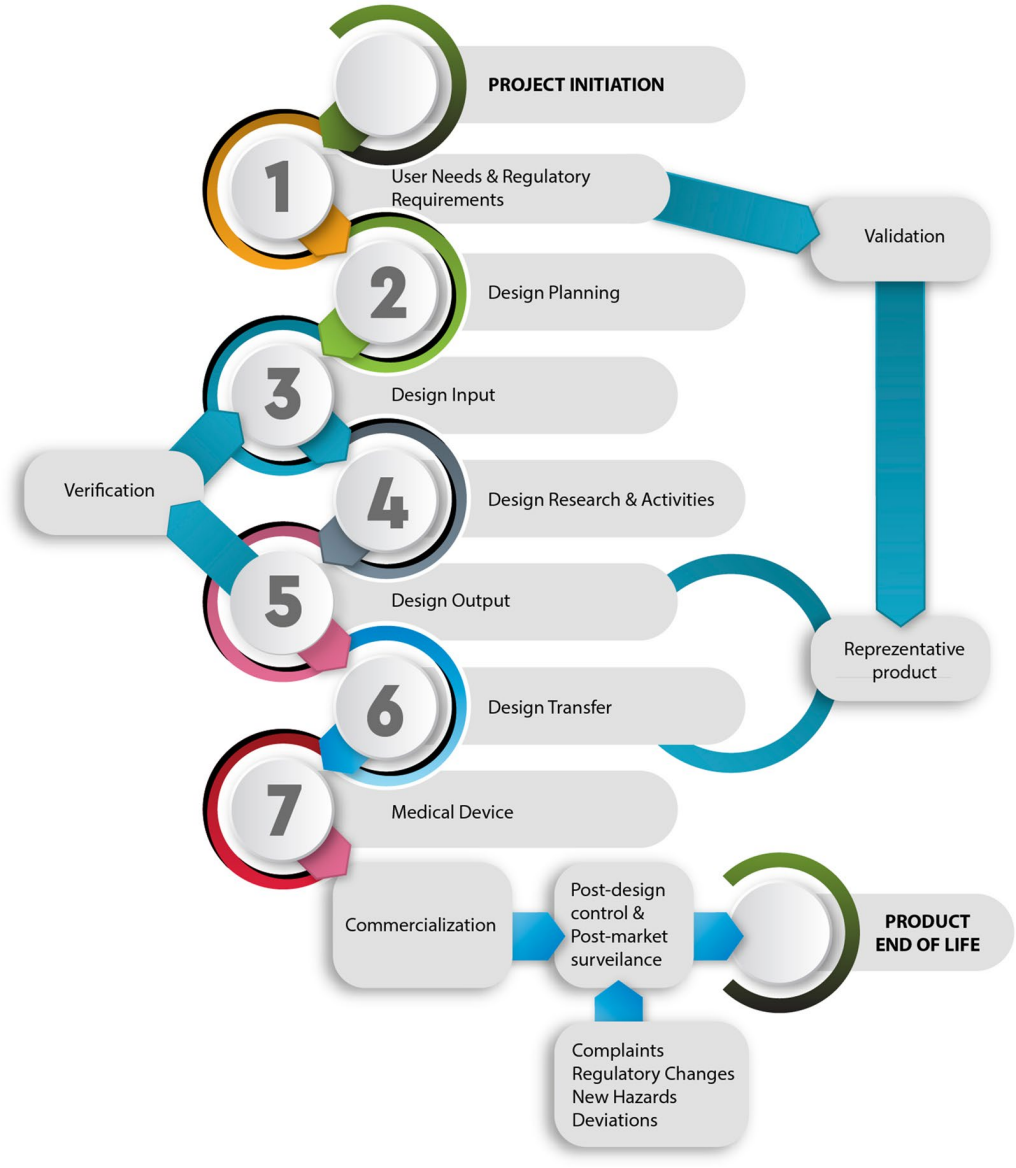
The Life-Cycle of a Medical Device.

On top of the above, MD design is an essential element of the device’s TPLC (Fig. 1) because it specifies both its functional safety and usability, therefore enables containment of error-prone processes. A product with high usability will make an MD less susceptible to use/user errors, and therefore easier to use [26, 59–62]. This is why MDR 2017/745 addresses Risk Management (RM) and requires evidence of validation of reduced risk based on usability testing [53, 63, 64]. If we consider the reported rates of adverse events attributable to design faults related to MDs’ user interface (UI) [65–69], it becomes obvious why design is essential in a setting where humans are expected to coordinate with MDs and not subjugate them.

The concept of safety and need for usability control has a concrete and very practical projection within the medical industry, with numerous examples of how poor design may result in device recalls and most importantly in exposing patients to injury or even death [13, 70–73]. The case of Pelonomi Hospital in South Africa is a clear example of how a design failure may lead to an urban legend [74]. When every Friday morning the occupant of a particular ICU bed was found dead, after excluding numerous logical explanations, such as bacterial infections, the nurses concocted the lethal bed story. Only later was it revealed that the janitor would unplug the life support equipment while cleaning the floors, accidentally killing the patient in the process. Although the life support equipment was performing as intended, failure to include any warnings against unplugging or alarms alerting to equipment disconnection from the power source resulted in patients’ death.

Apart from exposing patients to risk, poor design may also induce inconvenience for the user when, for instance, it becomes difficult to access the more frequently used functions of an MD because its actual use proved different from the one perceived by the Manufacturer. Rajkomar et al. [75] studied how nurses interact with computer-based infusion pumps in an ICU setting, observing how the cumbersome interface’s menu interfered with the timely infusion of proper volumes. A different set of problems may arise due to common errors that healthcare professionals have been ‘trained’ to ignore. Furniss et al [76]. evaluated the ergonomic characteristics of an in-house blood–glucose meter and highlighted the ease in accumulating a number of everyday errors (e.g., failure to display patient details, allowing more blood to be drawn during measurement, difficulty to access blood stripes, etc.) that eventually require a significant amount of time to correct, thus making the device obsolete, or result in patient endangerment.

A large number of MDs currently used for critical patient monitoring may also be affected by design errors (particularly poorly designed device interfaces), causing patient harm. MDs increasingly rely on software and even minor software changes/defects may have important implications for device functionality and clinical performance. Ronquillo et al. [77] identified all software defects-related MD recalls from 2011 to 2015. Among others, high‐risk software‐related recalls involved anesthesiology devices, such as ventilators and clinical decision support systems, with report details indicating that software shortcomings could result in a premature stoppage of mechanical ventilation. Recalls of infusion pumps intended to administer fluids to patients were also linked to software defects resulting in severe impairment of medication and fluids infusion [78–81]. The authors assert that having over 190,000 software units subject to high-risk recalls sets up a negative precedent, further aggravated by the impact of software such ABACUS TPN, which is intended for sustained use for large segments of the population without intermediate controls.

As we show, MD design allows potential problems to be identified and addressed during the design phase. MDs, when developed without considering the complex user-device-system relationships, become vulnerable while trying to adequately meet user requirements, potentially proving unsafe and ineffective in the real world, whether in a clinical setting or for independent patient use.

## Overview of Design-Related Regulations

**Figure 2.**
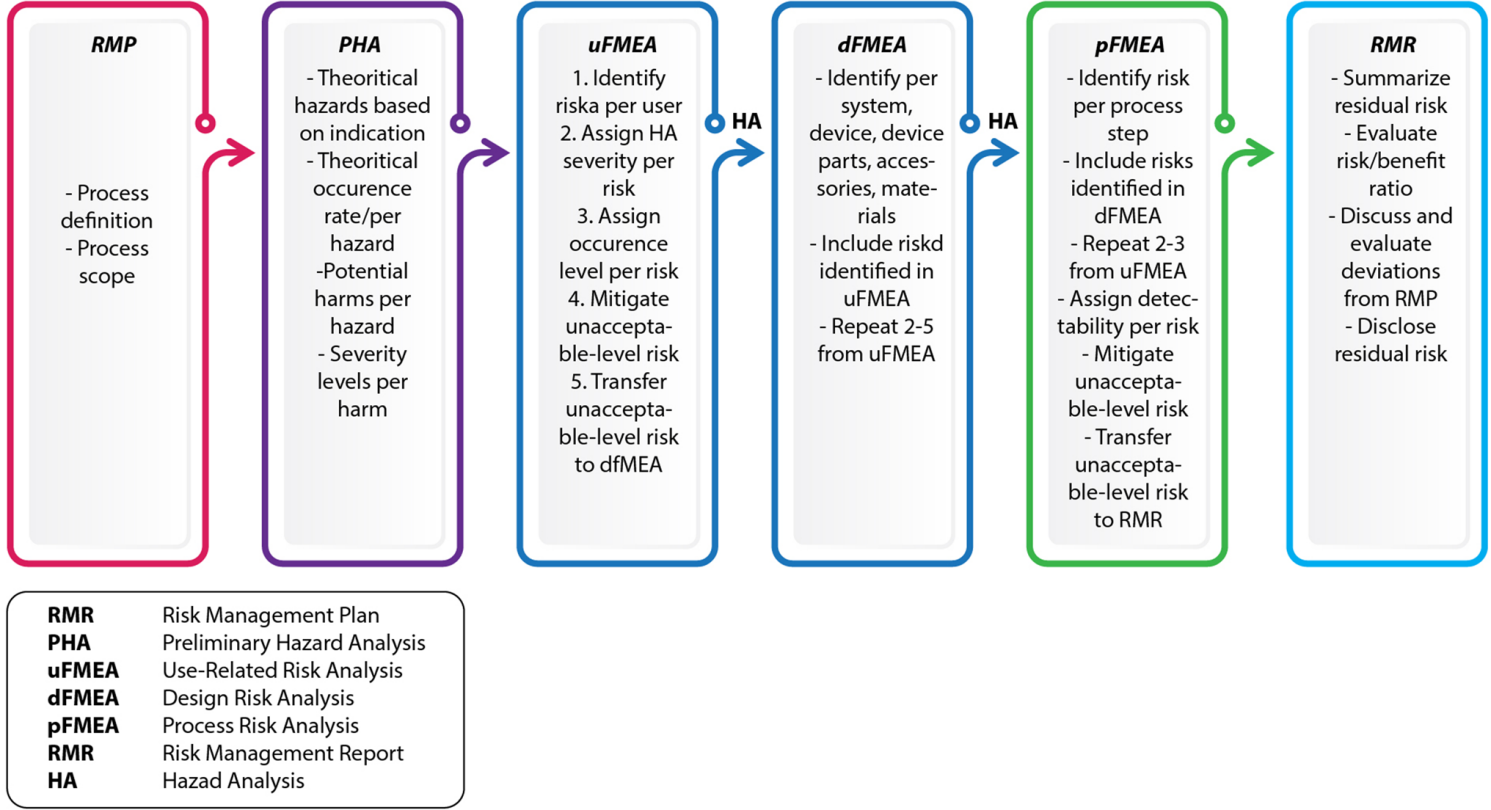
Risk Management Process During the Life-Cycle of a Medical Device.

Following conceptualization of an MD, design is crucial as a compromised design may impact the effectiveness and safety of the final product [19, 20, 53, 82, 83]. During this stage, MD design control is performed as part of the QMS requirements [35, 37, 53, 55]. In practice, an MD’s design aims to define the necessary specifications and exclude all potential hazards related to the intended use through the risk assessment process and conformity with national and international safety requirements [31, 33, 35, 36, 38]. Safe and effective MD design therefore begins as early as the product definition phase, even before product requirements and architecture have been specified [83]. Typically, hazards are identified through hazard analysis performed over the available architectural description of the intended MD and its operating setting. The likelihood and severity of identified hazards are then evaluated; all subsequent architectural design decisions are made based on the necessary mitigation strategies (Fig. 2). This dynamic process evolves throughout the TPLC of an MD [35, 84]. The ‘basic’ hazards that should at a minimum be evaluated in an MD device subsume (i) raw materials and wastes (e.g., toxicity, flammability, etc.), (ii) environmental factors (e.g., sensitivity to weather conditions), (iii) mechanical or electronic hazards, and (iv) user device interface hazards typically associated with HF (e.g., ineffective delivery, control of life-sustaining operations, etc.) [11, 85, 86].

MD design control is currently regulated by the updated ISO 13485:2016 and National and International guidelines such as FDA 21 CFR, Part 820 and MDR 2017/7454 [47, 87], which, while varying in scope, history, and phrasing, interrelate in regulating QM procedures used to corroborate intended performance and risk reduction for an MD (Table 1). Clause 7 of the updated ISO 13485 [88], in partial harmonization with MDR 2017/745, which actually defines more concrete requirements on MD’s post-market surveillance (PMS), sets Risk Management as a prerequisite during the product development stage, meaning that manufacturing practices (e.g., traceability of design inputs and outputs), Manufacturer infrastructures, and human resources are taken into account while producing a safe and effective MD.^1^ Similarly to FDA, who requires design controls for all Class II and III MDs and even some Class I devices (especially those classified as automated MDs with software), ISO 13485:2016 mandates design controls by redefining and expanding the purpose of Risk Management as the *[…]systematic application of management policies, procedures and practices to the tasks of analyzing, evaluating, controlling and monitoring risk[…]* [88].

**Table 1.** Overview of Design Control-Related Processes in ISO 13485:2016 (Clause 7: Product Realization) and FDA 21 CFR 820.3 Equivalent Regulation with Reference to the Risk Management Process.

MD Design Process	ISO 13845:2016	FDA 21 CFR 820	Risk Management Activity	Risk Management Output
Design & development planning	7.3.2	820.30 (a), 820.30 (b)	• Identification of the intended use and its potential hazards • Risk management plan corresponding to the identified risks	• Preparation of the hazards list • Outline of the risk management plan
Design input	7.2.3	820.30 (c)	• Hazard identification • Risk estimation	• Preliminary or initial hazards analysis
Design output	7.3.4	820.30 (d)	• Risk estimation and evaluation • Design mitigations • Determination of essential outputs	• Fault tree analysis • Failure modes effects analysis (FMEA)
Design review	7.3.1	820.30 (e)	• Risk evaluation to determine risk acceptability	• Risk decisions • Justification of any residual risk
Design verification	7.3.6	820.30 (f)	• Traceability analysis test in normal and fault modes • V&V activities corresponding to the identified risks	• Traceability matrix • V&V test results
Risk management	7.1 (see also ISO 14971:2012 process)	820.30 (g)		
Design validation	7.3.7	820.30 (g), 820.70 (i)		
(Potential) design changes	7.3.9	820.30 (i), 820.70 (b)	• Re-assessment of existing and potential new hazards/risks	• Update of RM documentation
Design transfer from product development to manufacturing	7.38	820.30 (h)	• Processing of risk assessment • Final safety decisions	• FMEA • Risk summary report
Preparation of a design history file	7.3.10	820.30 (j)	• PMS and vigilance data surveillance	• Review of the MD’s documentation

Overall, the risk-based approach of ISO 13485:2016 is reflected in QM by specific requirements in the control of internal processes, outsourcing practices, (clause 4), validation of computerized systems and software (clauses 4 and 7), MD development, evaluation of the supplying chain (clause 7), and, what is even more important, in the prevention and management of post-production data management (clause 8). Expansion of the risk process from design and development to the entire QM and harmonization with requirements of Competent Authorities (CAs) is a useful tool for effective risk mitigation as it allows Manufacturers to proactively identify hazards or usability discrepancies and therefore implement comprehensive preventive actions and eliminate sources of non-conformities.

Currently, the efficiency of pre- and post-approval surveillance systems for MDs is vividly debated in light of numerous ambiguous reactions of the Authorities to safety concerns involving orthopedic products [70], breast implants [10, 12, 13], and birth control implants [15]. Regulatory Authorities are intended to continuously assess the cost–benefit ratio of an MD, but there seems to be a hazard-causing gap between decision-making during the market authorization process and the PMS period, which, in some cases, allows design and Risk Management failures to go unnoticed until patients or users have been exposed to hazard. Harmonization of National and International Regulations could serve as a safety net that would ensure patients and users throughout the world have access to the same level of design and safety controls. This might be able to prevent situations such as the PIP breast implants scandal or the DePuy MoM hip replacement recalls [9, 34, 38].

This divergence of time and response severity is exactly why MD safety and compliance to standards is regulated as early as the design phase. The recent EU Regulation [47] takes the above-mentioned parameters into consideration and tries to adopt a more integrated risk-based approach, thus only partially aligning its General Safety and Performance Requirements with the corresponding standard for Risk Management [89]. In effect, design-related requirements, and by consequence Quality Management of an MD’s development, are now linked with the Risk Management process.

Annex I of MDR 2017/745 details requirements for Risk Management during MD design by setting several prioritized actions that must be implemented each time an MD moves to a new developmental stage. These tasks include risk elimination or reduction as far as possible to ensure safe design and manufacture, adequate protective measures against risks that cannot be eliminated, and provision of sufficient information, disclosure of residual risks, and user training to eliminate human-related errors. On the other hand, although the main content of ISO 14791 has not been altered, its new 2012 version deviates from MDR on risk reduction and treatment of negligible risks through annex ZA. Clause 3.4 of the revised ISO 14791 introduces the notion of risk reduction *as low as reasonably practicable* (ALARP). Obviously, the ALARP concept of risk reduction bears an inherent consideration of the economic burden on the Manufacturers when Authorities request risk reduction *as far as possible*. Therefore, providing an adequate MD design, addressing negligible and residual risks as early as phase I of development, is a strategy allowing Manufacturers to innovate while conforming to both MDR and ISO 14971. Being able to apply what clause 6.2 of the revised ISO 14971 refers to as *inherent safety by design*, takes hazards out of the equation at source, thus potentially reducing the overall risk burden of an MD and consequently its life-cycle cost and post-impaired post-market vigilance profile.

Within this context, the development and establishment of strategic Coordinated Registry Networks (CRNs), which will serve as a demonstration of the National Evaluation System for Medical Devices (NESMD), has been suggested [90]. The ongoing development of CRNs for orthopedic and vascular medical devices [91–93] is a characteristic example of this approach. In fact, FDA has repeatedly described its “vision” to incorporate CRNs into NESMD so as to reinforce MD post-market monitoring in a way that timely identification of post-market warning signals will be translated into a facilitator of premarket MD clearance that will allow the timely management of design defaults and previously unidentified hazards. Obviously, the success of such a venture depends on an active transformation of the contemporary MD landscape of wariness into a culture of good will and public exchange of information, while its standardization and cost-effectiveness can only be attained via the unimpeded cooperation of all stakeholders. Nevertheless, bridging the heterogeneity arising from disparate data sources with a reporting mentality could result in a dynamic, sustainable integrated evaluation of safety and performance data [94].

## Human Factors Engineering in MD Design

**Figure 3.**
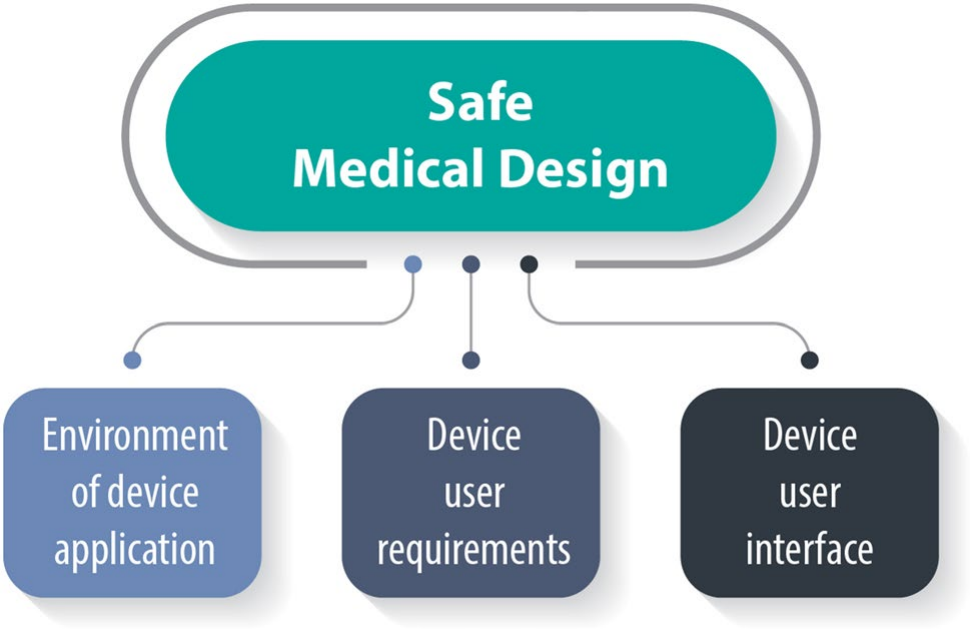
Human Factors Considerations.

The increasing need to ensure safety of both the patients and the healthcare professionals, as well as effective and efficient use of every device (Fig. 3), has led to the introduction of HFE principles and methods into the process of MD design and development, making them a key player, as reflected in ISO 13485 [88], which includes them in the QM process as design and development inputs.

FDA [95] defines HFE (also known as ergonomics, human engineering or usability engineering) [96, 97] as *[…]the application of knowledge about human behavior, abilities, limitations, and other characteristics of MD users to the design of MDs, including mechanical and software driven user interfaces, systems, tasks, user documentation, a user training to enhance and demonstrate safe and effective use.[…]*

Therefore, HFE requires that Manufacturers consider the user and the context in which the MD will be used. As already discussed, failure to address these aspects introduces safety hazards, resulting in use-error-related adverse events and UI-triggered MD recalls [98]. Conversely, taking a HF approach to MD design and development has been shown to have multiple benefits on patients, both by increasing patient safety [99, 100] and by enabling compliance with treatment [19], thus resulting in better health outcomes [101, 102]. Both patient and user satisfaction have been impacted by the implementation of HFE in MD design [101], while MDs ignoring HFE principles have been related to patient dissatisfaction and reduced compliance [62, 102, 103]. Sharples et al. [19] corroborated these findings for the acapella^®^ pulmonary embolism prevention device among adolescents with cystic fibrosis, while Herring et al. [104] showed that a user-oriented approach significantly contributed to increased surgeon comfort and satisfaction with laparoscopic surgical tools. Additionally, adopting HFE in MD design facilitates the identification and tackling of usability issues in early development stages, thus preventing expensive design changes further down in the development process, or after MD launch, thus reducing the chances of recalls [44, 105].

**Figure 4.**
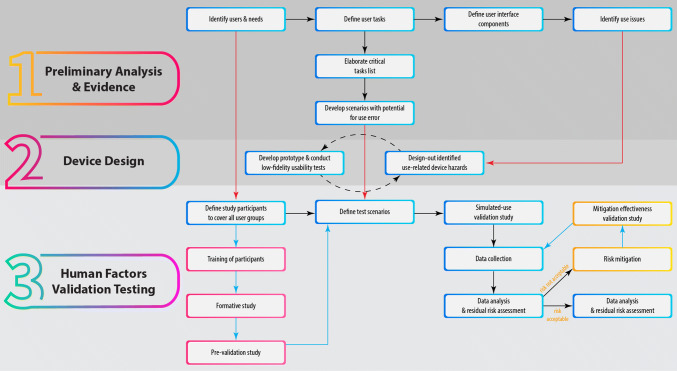
The HFE Approach for MD Design and Validation. The Necessary Procedures are Highlighted with a Blue Frame, While the Optional Processes, Which Depend on the Device or Results of the Use Validation Study, are Highlighted with Pink and Yellow Frames.

The use of HFE in MD design involves a multi-step process [78, 87, 97, 100], from definition of the users and context, to design and validation testing, as outlined in Fig. 4. Some of these processes are essential to successfully apply an HFE approach. Among them, the principle of proper identification of users is not always implemented, as it has been shown that key personnel within MD companies often replace the user of a device with the people who have the buying decision within a healthcare institution and who are rarely the end users [97]. Definition and classification of tasks is the next critical phase, ranking them based on likelihood of occurrence, severity, and probability of escaping detection [106]. Critical tasks, unless properly performed, may result in serious harm to the user [87]. Scenarios for potential use error are then developed and represent the basis for the test scenarios of the HF validation testing stage [100]. Subsequently, defining the UI allows identifying potential use-related issues from the first stages of the design process, especially when compared with similar MDs [87]. The main goal throughout the process is to design-out as many use-related hazards as possible [78, 107], while keeping in mind that re-designing might be necessary if new hazards are identified [87].

Once a prototype is available, HF validation testing is implemented after a careful definition of the user groups [100]. It is essential for all simulated-use tests to be carried out in conditions relevant to the real-life use of the device [67]. At this stage, a thorough residual risk assessment is required to identify any severe use-related errors and appropriate risk mitigation approaches [87, 106].

Regardless of the methods used throughout the HFE process, certain design principles apply to ensure the production of a safe MD [108, 109]: (i) consistency and standards, signifying that it should be evident to the users what the colors, layout, and words mean (e.g., red color for danger, ‘del’ for delete); (ii) visibility of system state, clearly informing the user about the state of the system, using appropriate displays and indicating possible future steps; (iii) match between system and world, meaning that the user’s mental model of what the system looks like fits with how the system actually presents itself; (iv) minimalism, i.e., not giving or requesting information that is not necessary for the proper functioning of the MD; (v) reduced memory load, meaning it should not be necessary for the user to memorize large amounts of information in order to successfully complete a task; (vi) informative feedback, by communicating to the user in a concrete and direct manner at every step, providing information about the user’s actions and their results; (vii) good error messages, which are precise and clear, delivered in an unambiguous language; (viii) prevent errors, insofar as possible, through safe-by-design interfaces.

Disregarding such principles leads to use errors even by highly trained medical professionals, as, for example, in electrosurgical units where device components are not grouped according to their function, interface symbols do not have commonly understood meanings and receptacles for accessory instruments also fit plugs not intended for them [67]. However, designing MDs according to HFE principles leads not only to safer products and an overall increased user satisfaction [20], but also to devices with a higher degree of usability, which translates into faster market access and increased speed with which a task is performed with the device versus without the device (i.e., efficiency). Overall, the HFE process has a risk-centric, three-stage approach (preliminary analysis, formative evaluation and design modification, validation testing) within MD design, aimed at preventing human errors by designing out characteristics that could lead to mistakes. This way, HFE is incorporated into MD design via Risk Management [65, 68].

## Concluding Remarks

Every piece of MD technology is part of and shapes the human-technology dipole, which is a unitary entity interacting in almost all medical actions and decisions. For this reason, enhancement of patient and physician safety requires an integrated approach of MD design, taking into consideration effective risk reduction as early as possible in an MD’s TPLC. The MD industry is currently under the influence of tailwinds due to the imminent full-scale implementation of MDR, which remains a challenge especially for SMEs, and recurrent safety-related scandals involving sophisticated MDs. Within this context, implementation of harmonized strategies, including the use of HFE, could serve as a headwind facilitating the introduction into the market of integrated solutions that will enhance healthcare provision. Survival of medium-size Manufacturers is critical for delivering innovation to patients and from a regulatory perspective, it is important to realize that patients’ access to new health technologies is not only affected by approvals of CAs but also by the potential of companies to invest into and establish a risk-based QMS from concept through manufacturing and into the field. For years, cost of innovation and fear of stringent audits have detained the MD ecosystem from developing a self-evaluation process, which would be able to monitor and account for the safety of users and patients in real time. Incorporation of HFE in design control, risk-based approach of TPLC, implementation of the latest Regulations, and further development of Registry Networks are setting a path towards the right direction, i.e., taking patient safety into the hard core of an MD’s regulatory cycle.

## Footnotes

1 ISO13485:2016; Clause 7.1: *[…] The organization shall document one or more processes for Risk Management in product realization. Records of Risk Management activities shall be maintained […].*
